# Towards an Integrated Multi-Omic Approach to Improve the Diagnostic Accuracy of Fine-Needle Aspiration in Thyroid Nodules with Indeterminate Cytology

**DOI:** 10.3390/diagnostics15121506

**Published:** 2025-06-13

**Authors:** Monia Bordoni, Nairus Aboud, Francesca Silvetti, Augusto Taccaliti, Giancarlo Balercia, Gianmaria Salvio

**Affiliations:** Division of Endocrinology, Department of Clinical and Molecular Sciences, Università Politecnica delle Marche, Via Tronto 10/A, 60126 Ancona, Italy; monia.bordo@gmail.com (M.B.); drssa.n.aboud@gmail.com (N.A.); f.silvetti@ospedaliriuniti.marche.it (F.S.); a.taccaliti@univpm.it (A.T.); g.balercia@staff.univpm.it (G.B.)

**Keywords:** thyroid cancer, follicular thyroid neoplasm with papillary-like nuclear features, follicular adenoma, genomics, proteomics, metabolomics, mass spectrometry, spectroscopy

## Abstract

Thyroid nodules are accidentally found in up to 68% of people undergoing neck ultrasound (US) examination, and fine needle aspiration (FNA) is the current gold standard to discriminate between malignancy and benign lesions. Unfortunately, one-third of FNAs are classified as indeterminate, requiring surgery for definitive diagnosis. This leads to high costs and health risks of unnecessary procedures, since malignancies are observed in less than half of operative specimens. This narrative review aims to describe the most innovative multi-omics approach techniques, including genomics, proteomics, and metabolomics, aimed at making the preoperative evaluation of indeterminate thyroid nodules more accurate. The advantages and disadvantages of the techniques are described in detail, and a SWOT (strengths, weaknesses, opportunities, and threats) analysis of the multi-omic approach is provided.

## 1. Introduction

Thyroid nodules are a very common occurrence in the general population, being detected in up to 68% of unselected individuals using high-resolution ultrasound (US) evaluation [1]. When a thyroid nodule is incidentally discovered, exclusion of a thyroid malignancy is key; indeed, malignancies only constitute a minority (7–15%) of US-detected thyroid lesions, but the therapeutic approach is different compared to benign lesions, often requiring partial or total thyroidectomy [1]. Remarkably, up to 50% of thyroid nodules can be identified as highly likely to be benign on US and may be followed by periodic sonographic monitoring; however, the detection of higher-risk US features such as microcalcifications, hypoechoic parenchyma, irregular shape or borders, and taller-than-wide shape warrants further investigation [1]. Because of its low cost and high effectiveness, fine needle aspiration (FNA) is the current gold standard to exclude the malignant nature of a thyroid nodule, therefore guiding patients’ stratification and management towards surgical or conservative approaches. Nevertheless, about one-third of FNAs are classified as indeterminate for malignancy, thus requiring surgery for a definitive diagnosis [2]. In greater detail, the Bethesda System for Reporting Thyroid Cytopathology has two categories of indeterminate cytology: atypical or follicular lesion of undetermined significance (Bethesda III) and follicular neoplasm/suspicious for follicular neoplasm (Bethesda IV), each accounting for 10% of FNA results. The observed rates of thyroid tumor after surgical excision in these categories range from 6% to 48% for Bethesda III and 14% to 34% for Bethesda IV [3]. This poses an important problem in terms of standardizing treatments, preventing unnecessary invasive procedures, and reducing healthcare costs. The greatest challenges for a cytopathologist are the distinction between follicular/oncocytic adenoma versus follicular/oncocytic carcinoma and between non-invasive follicular thyroid neoplasm with papillary-like nuclear features (NIFTP), recently introduced as a “low risk neoplasm”, versus papillary thyroid carcinoma (PTC), as they are largely based on architectural histopathological features [4]. It is therefore key to find powerful diagnostic tools to assist cytopathologists in the stratification of malignancy risk and to guide towards the most appropriate therapeutic management of indeterminate thyroid nodules. Over the last decades, the role of molecular analysis has been extensively explored, and several molecular-based high-throughput techniques have been proposed to improve the pre-operative risk assessment of indeterminate thyroid nodules. Improving the diagnostic power of US with conventional imaging techniques such as magnetic resonance imaging (MRI) and computed tomography (CT) has recently been proposed, but available data are scarce [5,6]. The aim of this review is to summarize such recent advances in the evaluation of indeterminate thyroid nodules, ranging from the application of standard molecular testing to the more complex study of the genomic, proteomic, and metabolomic signature of thyroid nodules.

## 2. Genetic Testing: Breakthroughs and Challenges of “Rule-In” and “Rule-Out” Tests

**Figure 1:**
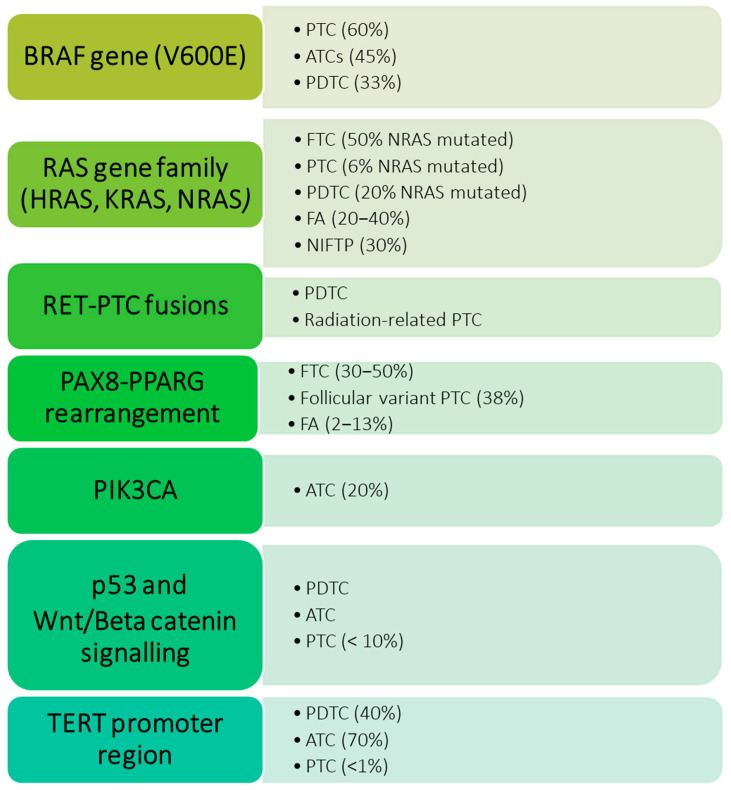
Genetic alterations are most frequently observed in neoplastic proliferations of the thyroid, and their incidence rate varies in different types of lesions. PTC: papillary thyroid carcinoma; FTC: follicular thyroid carcinoma; FA: follicular adenomas; NIFTP: non-invasive follicular thyroid neoplasm with papillary-like nuclear features; ATC: anaplastic thyroid carcinoma; PDTC: poorly differentiated thyroid carcinoma.

There are several genetic alterations that have been strictly correlated with thyroid tumors. Mutations of the BRAF gene, more commonly the V600E mutation, are found in up to 60% of PTCs [7] while mutations in the RAS gene family (HRAS, KRAS, and NRAS) are reported in follicular thyroid carcinomas (FTCs), benign lesions such as follicular adenomas (FA), and low-risk neoplasms as the NIFTP [8,9,10]. Other genetic alterations are RET-PTC fusions, commonly found in radiation-related PTC [11], and PAX8-PPARG rearrangement, which, similarly to RAS gene mutations, is linked to follicular benign and malignant lesions [12]. Lastly, PIK3CA, TP53, Wnt/Beta catenin signaling, and TERT promoter region mutations have been reported in aggressive thyroid cancers such as anaplastic thyroid carcinoma (ATC) and poorly differentiated thyroid carcinoma (PDTC) [13]. Figure 1 summarizes the main gene mutations found in different types of thyroid proliferations [7,8,9,10,11,12,13,14,15].

Therefore, while the recognition of certain specific genetic alterations such as BRAF^V600E^ has a high positive predictive (PPV) value for malignancy (for BRAF^V600E^ 98%), this is not the case for many other common mutations that can be found with variable rates even in benign thyroid lesions, such as HRAS^Q61R^, NRAS^Q61R^, BRAF^K601E^, and the PAX8/PPARG and RET/PTC rearrangements [16,17]. In 2014, the Cancer Genome Atlas Research Network studied the molecular profile of 496 classical and follicular variant PTCs and described two molecular subgroups with distinct genomic, epigenomic, and proteomic profiles: BRAF^V600E^-like and RAS-like [7]. Remarkably, this project mapped a significant number of mutations that subsequently led to the creation of several panels and platforms that have been introduced in clinical practice.

In more depth, starting from 2009, several molecular panels comprising multiple genes have been developed to assist in the assessment of the malignancy risk of thyroid nodules and to improve the diagnostic accuracy of FNA [18]. These molecular tests are particularly important due to their potential impact on the stratification of patients and management of thyroid nodules with indeterminate cytological classification. Importantly, the ability of a molecular test to “rule in” or “rule out” cancer depends on its PPV and negative predictive value (NPV), respectively. Diagnostic tests featuring high sensitivity and high NPV are considered good tests to “rule out” the presence of disease, therefore, a negative test strongly advises a conservative approach. On the other hand, diagnostic tests with a high specificity and high PPV are good to “rule in” disease, meaning that a positive test strongly points towards the presence of a cancerous lesion and the necessity for an invasive approach. While an ideal test would display both high sensitivity and specificity, thus providing excellent accuracy at distinguishing between benign and malignant lesions, in practice, an increased sensitivity of a test reduces the specificity, and vice versa.

The first molecular test, developed in 2009 by Nikiforov YE et al. [19] through a prospective study, was a seven-gene panel designed to detect point mutations in the BRAF, KRAS, NRAS, and HRAS genes (using real-time LightCycler PCR and fluorescence melting curve analysis), as well as RET/PTC1, RET/PTC3, and PAX8/PPARG rearrangements (identified from RNA by RT-PCR with primers flanking the respective fusion junctions). These genetic alterations had previously been reported in the literature as the most frequently associated with differentiated thyroid carcinomas. However, prior studies were limited by their retrospective design and focused on the detection of individual genetic alterations, without evaluating their application in a clinical setting [19]. This seven-gene-panel test demonstrated high specificity (99% and 97% for Bethesda III and IV nodules, respectively) and positive predictive value (88% and 87% for Bethesda III and IV nodules, respectively), but it was limited by low sensitivity (63% and 57% for Bethesda III and IV, respectively). The seven-gene specific mutation panel has therefore been approved as an efficient “rule-in” test for thyroid malignancy based on its high specificity and PPV, but it is limited due to the restricted number of analyzed genes [18].

Thus, in 2012, the Afirma Gene Expression Classifier (GEC, Veracyte, San Francisco, CA) was introduced with the intent of providing a “rule out” type of test to exclude malignancy and reduce unnecessary thyroid surgery based on RNA profiling [20,21]. Afirma GEC measures the expression of 167 genes and shows a sensitivity of 92%, a specificity of 52%, and an NPV of 95% and 94% for Bethesda III and IV nodules, respectively, when predicting on a set of 265 FNAs with indeterminate cytology [20,21]. More recently, Afirma has published a genomic sequencing classifier that exploits next-generation RNA sequencing and whole transcriptome analysis, combined with machine learning, to improve the test specificity and further reduce unnecessary invasive procedures. This has, indeed, an improved specificity of 68%, sensitivity of 91%, NPV of 96%, and PPV of 47%. This classifier, despite remaining a rule-out test, showed an improved “rule in” ability compared to the Afirma GEC [22].

From 2013 to 2015, the ThyroSeq multigene genomic classifier (GC) was developed and updated in several versions (ThyroSeq v1, v2, v2.1, v3) as a comprehensive test to concomitantly “rule in” and “rule out” thyroid malignancy [23,24,25]. This test is based on next-generation sequencing (NGS) and the ThyroSeq v3 GC analyses the DNA and RNA of 112 thyroid cancer-related genes for point mutations, insertions, deletions, gene fusions, copy number alterations, or gene expression alterations, with a reported sensitivity of 94%, specificity of 82%, NPV of 97%, and PPV of 66% on a cohort of 247 FNAs with indeterminate cytology. The improvement in sensitivity and NPV of this test was obtained by testing for more pathogenic mutations compared to the previously used tests [26].

The other commercially available test with high PPV and NPP is the ThyGeNext/ThyraMIR, a multiplatform test allowing for the evaluation of genetic alterations of DNA, mRNA (38 common gene fusions), and gene expression regulators known as microRNA (10 genes). This is also proposed as a powerful test able to both “rule in” and “rule out” malignancy. In greater detail, the test showed a sensitivity of 89%, with 85% specificity, 68% PPV, and 97% NPV for Bethesda III nodules and 91% for Bethesda IV nodules [27].

Despite the considerable heterogeneity between these techniques and their variable results, they can be considered valuable tools to better characterize the nodules of indeterminate cytology and to reduce the number of unnecessary procedures. To date, the analysis of genetic panels represents the most advanced ancillary method supporting thyroid cytology. Nevertheless, its routine implementation in clinical practice remains limited due to the restricted availability of high-throughput technologies and the associated high costs, variable from $3000 to $6400 per test [28]. In this context, individual centers have progressively developed in-house, non-commercial assays targeting a limited number of genes and characterized by relatively low costs. Owing to their good specificity, these rule-in tests have proven to be useful in providing additional information to conventional cytopathology in clinical practice [29].

## 3. Study of Proteomic Signature: From Point-of-Care Protein Panels to Neural Networks

**Figure 2:**
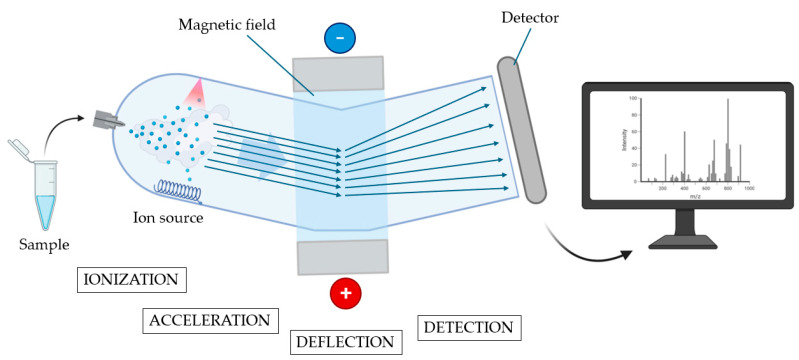
Principles of mass spectrometry. In the first phase (ionization), the vaporized sample is ionized, and the ions generated are accelerated through an electrical field (acceleration phase) and reach a magnetic field. In the deflection phase, ions are deflected by the magnetic field according to their mass and charge. The subsequent flow of ions reaches a detector to be amplified and recorded (detection phase). All the received information is subsequently elaborated and presented in the form of a mass spectrum (created using BioRender.com) (accessed on 31 May 2025).

Another feasible way that has been explored to improve the pre-operative risk assessment of malignancy in indeterminate nodules is the study of protein expression; this is performed through the definition of proteomic signatures that could allow for the distinction between benign and malignant lesions. A remarkable tool that enabled this type of study is mass spectrometry (MS), an analytical technique that is able to sort and identify a wide range of clinically relevant molecules within complex samples. In greater detail, MS is able to separate ionized particles as atoms, molecules, and clusters by using differences in their mass-to-charge ratios. MS consists of three main components. The ion source creates electrically charged particles from the sample molecules by exploiting high-energy sources as a high-energy electron beam (electron ionization), a high-energy electron beam in gas with chemical reactions (chemical ionization), ultraviolet light (photoionization), laser beam (matrix-assisted laser desorption: MALDI). The second component is the mass analyser, which separates the ions according to their mass-to-charge ratios through the application of electromagnetic fields; there are many types of analysers, but the most commonly used are quadrupoles, magnetic sectors, and time-of-flight mass analysers. The third component is the detector, which records the signals from the ions and sends them to a computer that calculates the abundances of each ion, creating a mass spectrum. Fundamentally, the mass spectrum is a graph displaying the mass-to-charge ratio on the x-axis and the relative intensity—corresponding to the number of detected ions—on the y-axis. Different elements of the sample correspond to different peaks [30] (all the steps are illustrated in Figure 2).

A first attempt at differentiating the nature of thyroid nodules based on post-operative FNA with MS was performed by Giusti and colleagues in 2008 [31]. They studied protein expression of FNA of PTC and normal tissue samples using two-dimensional electrophoresis and matrix-assisted laser desorption ionization time-of-flight/MS (MALDI-TOF/MS). This led to the identification of 17 protein that are up-regulated in classical variant PTC and/or tall cell variant PTC with respect to controls; these proteins included transthyretin precursor, ferritin light chain, proteasome activator complex subunit 1 and 2, alpha-1-antitrypsin precursor, glyceraldehyde-3-phosphate dehydrogenase, lactate dehydrogenase chain B, apolipoprotein A1 precursor, annexin A1, DJ-1 protein, and cofilin-1 [31]. Based on these results, they then used Enzyme Linked Immunosorbent Assay (ELISA) and Western blot (WB) assays to test the levels of five different proteins that were found differentially expressed (L-lactate dehydrogenase B chain, Ferritin heavy chain, Ferritin light chain, Annexin A1, and Moesin) on pre-operative FNA samples. ELISA and WB analysis confirmed the increase in lactate dehydrogenase heavy chain, Moesin, and Annexin in pre-surgical FNA of PTC, compared to controls. This study was the first to demonstrate the similarity of the proteomic signature of pre-surgical FNA and FNA made on surgically removed tissue. This is relevant, as it paved the way for subsequent studies that only used pre-surgical FNA for the definition of the malignancy of undetermined nodules [32].

The innovations were not only limited to the possibility of using pre-surgical samples, thus potentially eliminating unnecessary surgery, but also in the advanced technologies that allowed for the study of single groups of cells. Indeed, while traditional MS requires the separation and purification of a sample and the loss of spatial information, MS imaging technologies have been employed to discriminate between benign and malignant nodules based on the analysis of thousands of analytes and their spatial distribution. The very first preliminary study to explore the application of MS imaging in thyroid cytopathology was conducted by Mainini and colleagues in 2013 [33]. They applied Matrix-assisted laser desorption ionization/imaging MS (MALDI-IMS) to integrate proteomic data into the morphological classification of FNAs. MALDI-IMS is a unique technology that enables the evaluation of the spatial distribution of biomolecules in groups of cells, thus combining both the molecular and the morphological characteristics of a cytological specimen. Mainini et al. evaluated different cytological samples, including hyperplastic nodules, classical and follicular variant papillary carcinomas, and Uncertain Malignant Potential tumors, demonstrating a relevant difference in the molecular signature between the benign and malignant proliferations [33]. Pagni and colleagues provided complementary data, using the same technology, to differentiate between hyperplastic proliferations and clonal follicular lesions (oncocytic adenoma, medullary thyroid carcinoma, and classical variant PTC), detecting a panel of six differentially expressed proteins. Interestingly, the proteomic profile of a hyperplastic area of a patient with a follicular lesion was superimposable with that of a purely hyperplastic nodule, thus highlighting the ability of this technique to investigate different areas on the same cytological sample [34]. In a subsequent study, they then demonstrated the possibility of correlation between a putative “proteomic classification” based on protein profiles with the histological classification, thus providing a model that could pre-operatively predict the nature of the lesion. This model also has the ability to identify specific signatures of different environments within the benign lesions as hyperplastic proliferation, FA, and Hashimoto thyroiditis [35]. The same group later introduced a novel approach to create a proteomic diagnostic tool in thyroid cytopathology, always using the MALDI-MSI technology [36].

Proteomic-based techniques could become relevant tools in the definition of malignancy from FNA samples, allowing for the elimination of many unnecessary procedures. An even more powerful combination could be the concomitant evaluation of genomic and proteomic data. In 2020, Sun and colleagues presented the first protein-based artificial neural network classifier that could facilitate the classification of thyroid nodules. This is a thyroid-tissue spectral panel to be potentially considered as a future point-of-care diagnostic test, based on MS, to complement immunohistochemical data and nucleic acid-based test [37]. This was then perfected and validated in a second study published two years later, which examined a larger number of thyroid proteome profiles [38].

## 4. Thyroid Nodules Metabolic Signatures: From Local to System-Level Screening for Thyroid Malignancies

Another key tool to discriminate the malignancy risk of a nodule could be the analysis of metabolites. Alterations in cancerous thyroid cells are associated with variations in their metabolic byproducts, which can be observed through different molecular-based high-throughput techniques. These alterations have been described for the first time by Dehoog and colleagues, who used desorption electrospray ionization mass spectrometry (DESI-MS) imaging, a powerful technique that allows for the detection of small molecules such as lipids, fatty acids, and metabolites directly on tissue samples [39]. They used DESI-MS on 206 banked human thyroid samples to determine the different molecular signatures of normal thyroid tissue, FA, FTC, and PTC, and to consequently build two statistical classification models: benign versus PTC and benign versus FTC. Then, DESI-MS imaging was obtained from clusters of thyroid cells collected by FNA samples, and the predictive models built from tissue samples were assessed. The benign versus PTC showed a sensitivity of 96% and a specificity of 91%, whereas the benign versus FTC model showed a sensitivity of 100%, a specificity of 88%, and an overall accuracy of 89%. In the benign versus PTC model, several lipid species, including phosphatidylinositol (PI), have been found to be differentially expressed, with PTC containing PI with higher degrees of saturation compared to normal tissue. Phosphatidic acid is also differentially expressed, with PTC expressing mono-unsaturated rather than polyunsaturated phosphatidic acids. In the benign versus FTC model, several metabolite intermediates were differentially expressed, and molecules such as malate, glutamate, and succinate were selected as FTC-characterizing metabolites. Interestingly, these models are in line with the nodule stratification evaluated through the Afirma GEC [39].

Metabolic profiling was also performed by combined gas chromatography−time of-flight MS (GC−TOFMS) and ultraperformance liquid chromatography−quadrupole time-of-flight MS (UPLC−QTOFMS) by Xu and colleagues in 2015 [40]. The combination of the two MS platforms provides very high sensitivity and selectivity, and was used to obtain metabolic information that could distinguish PTC from thyroid adenoma (TA). Interestingly, several similarities in the metabolic signatures were detected between PTC and TA compared to normal thyroid tissue, as increased glycolytic activity, amino acid metabolism, one-carbon metabolism, tryptophan metabolism, and TCA cycle intermediates, suggesting that these two types of proliferations share common metabolic pathways. Some differences in the molecular signature were also detected; in more detail, purine and pyrimidine metabolism, taurine and hypotaurine levels were higher in PTC compared to TA, while increased levels of fatty acid and bile acids were reported in TA [40].

Another tool used for metabolomic investigation is spectroscopy, which provides both quantitative and qualitative definition of the composition of a biological sample, through the identification of the different electromagnetic waves emitted or absorbed by its molecules. Different spectroscopic techniques are available depending on the method of generation and detection of electromagnetic radiation; however, as a general rule, the output is a spectrum in which different wavelength peaks correspond to specific biomolecules [41,42]. Therefore, applying multivariate statistical analysis to identify and compare the spectral fingerprints (“chemometric approach”) of various thyroid cell populations may yield valuable insights for the cytological assessment of indeterminate cases. To date, there is a scarcity of literature on spectroscopy studies related to thyroid pathology, particularly concerning the application of two spectroscopic techniques: Fourier Transform Infrared (FT-IR) Spectroscopy [41], and Raman Spectroscopy (RS) [43]. These are fast and low-cost techniques that allow for the acquisition of a spectral profile of a biological sample—either in solid or liquid form, and in small amounts—without specific pre-processing [41]. Infrared (IR) spectroscopy analyzes the interaction between IR radiation (12,800 to 10 cm^−1^) and molecular vibrations, particularly within the mid-IR range (4000–500 cm^−1^), where organic compounds show strong absorption [44]. Modern instruments typically employ FT-IR technology, which collects all wavelengths simultaneously as an interferogram, later converted into a spectrum via the Fourier Transform. The system uses an interferometer consisting of a light source, a beam splitter, mirrors, a laser, and a detector. The beam splitter directs the IR light into two separate paths, one toward a fixed mirror and the other toward a moving mirror. After reflection, the beams are recombined and transmitted through the sample, which absorbs specific IR frequencies, depending on the types of atoms and chemical bonds it contains. Indeed, absorption occurs only for radiation frequencies that resonate with the intrinsic vibrational modes of the molecules. The result is a detailed infrared spectrum, showing absorbance (Y-axis) versus wavenumber (X-axis), with the 4000–1500 cm^−1^ region used for functional group identification and the 1500–500 cm^−1^ range, known as the “fingerprint region”, providing compound-specific patterns [44].

**Figure 3:**
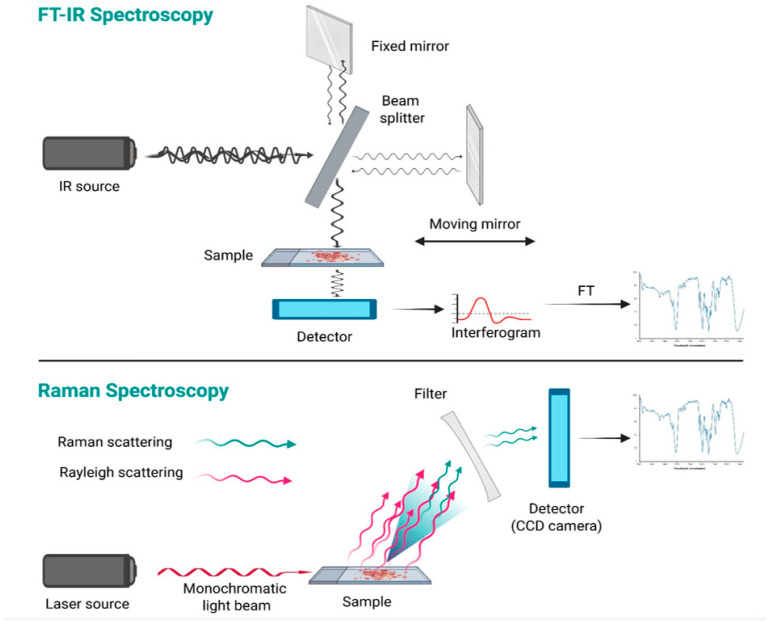
Principles of FT-IR and Raman Spectroscopy. Spectroscopy exploits the interaction of light with matter to obtain information about the structure or characteristics of a material. FT-IR spectroscopy is based on the detection of light absorbed by the sample, whereas FT-Raman Spectroscopy relies on the detection of scattered light (created using BioRender.com) (accessed on 19 April 2025).

As previously mentioned, the frequencies of a light beam that match the vibrational modes of a molecule are absorbed by the substance. The remaining light is scattered: the majority of the scattered electromagnetic waves retain the same energy and wavelength as the incident beam (elastic scattering, also known as Rayleigh scattering), while a small fraction of the scattered light (approximately 0.0000001%) undergoes a change in energy (inelastic scattering, known as Raman scattering) [45]. The identification of the frequency shifts between the incident light and the Raman-scattered light enables the determination of the specific molecular composition of the sample [45]. This is the principle of RS. By using a monochromatic laser with a known wavelength (typically 532 nm, 785 nm, 830 nm, or 1064 nm), part of the laser light is absorbed by the sample to excite molecular vibrations, causing Raman scattering. To eliminate Rayleigh scattering, a specialized optical filter is employed, and the remaining scattered light is captured by a CCD (charge-coupled device) detector, allowing for the determination of its frequency and corresponding Raman shift. Once the Raman shifts are known, this information is used to generate the Raman spectrum in which each peak in the spectrum corresponds to a specific frequency of light absorbed by the sample, similarly to FTIR spectroscopy [45] (principles of FT-IR and Raman Spectroscopy are illustrated in Figure 3).

Several studies based on FT-IR Spectroscopy have focused on the analysis of histological samples obtained post-thyroidectomy. From this investigation, alterations in lipid expression (within the spectral region of 4000–2500 cm^−1^ and at 1700 cm^−1^) and carbohydrates (within the region of 1500–600 cm^−1^) have been observed. These alterations have been attributed to an increased consumption by malignant neoplastic tissues [46,47,48,49]. Furthermore, differences in the expression of nucleic acids and proteins have also been identified [47,49,50], such as the presence of β-sheet structured proteins in malignant tissues (signal at the wavenumber of 1600 cm^−1^) [51]. On the other hand, the application of FTIR spectroscopy to thyroid FNA has been underexplored. One of the first studies on this topic was conducted by Schultz et al. in 1999 [52], who analyzed both cytological and histological samples from patients undergoing thyroidectomy. Specifically, the molecular composition of thyroid cells and the cell-free supernatant from 76 aspirates was studied, identifying four distinct spectroscopic clusters based on molecular expression patterns. They then hypothesized that these distinct patterns could also be detected through the analysis of the cell-free liquid derived from FNA. The possibility that the supernatant from aspirates might express the same metabolic products, and thus reflect the tumor’s fingerprint, could improve the detection of thyroid cancer in cases of insufficient cellular sampling during aspiration. From the spectroscopic study of histological tissues, a bivariate analysis was conducted, revealing increased expression of DNA (band at 968 cm^−1^) and reduced expression of proteins (band at 1550 cm^−1^) in neoplastic tissue, including MTC, with the exception of PTC, which exhibited a higher protein component. Ultimately, Schultz et Al. concluded that there was minimal overlap between the bivariate analysis histograms of neoplastic and normal tissues, allowing for a clear spectroscopic distinction between the two cellular populations; however, the definitive histotype of neoplastic tissues was not specified and a comparison between cytological and histological spectroscopic analyses was not delineated in this study [52].

Subsequently, Liu et al. performed spectroscopic analysis on 89 FNA cytological samples, comparing the results with definitive diagnoses (either cytological or histological) of the analyzed nodules [53]. The investigation included FTC in 2.2% of cases, PTC in 10.1%, and other types of carcinoma in 7.9% of cases. For the latter, the histotype was not specified, nor whether undifferentiated thyroid carcinomas or MTC were present. Through supervised linear discriminant analysis (LDA), the corresponding IR spectrum was assigned to each definitive diagnosis, identifying representative class average spectra (training set). Then, on the test set, the class assignment algorithm thus delineated demonstrated a good accuracy (90.2%) of IR spectra-based diagnoses. Finally, nine spectral regions (ranging from 1800 to 900 cm^−1^) were identified with the highest discriminatory capability between benign and malignant lesions, corresponding to the expression of various metabolic products such as lactic acid, side chains of proteins, lipids, carbohydrates, and DNA. Thus, the spectroscopic signature from DNA and other metabolic byproducts was adequate to achieve effective differentiation of the spectra.

Further studies on thyroid cytology have been conducted using RS [43]. Most of these studies have been carried out by De Oliveira et al.; however, they stem from research on single cells isolated from frozen or fresh tissue samples of thyroid nodules. The first study, conducted in 2019, compared the spectroscopic profile of cells obtained from fresh tissues of five PTCs and five benign nodules. This study revealed a higher expression in PTCs of spectral regions at 1080 cm^−1^ (phosphodiester group in nucleic acids), 1260 cm^−1^ (lipids), 1293 cm^−1^ (cytosine), 1430 cm^−1^ (lipids), and 1667 cm^−1^ (amide I), whereas benign cells exhibited greater expression of the bands at 1003 cm^−1^, 1031 cm^−1^, and 1362 cm^−1^ (phenylamine and tryptophan) [54]. In 2022 the same group, using spectroscopic classification on FNA samples from ex vivo thyroid nodules, achieved an accuracy of 84% in distinguishing between FA (*n* = 2) and oxyphilic cell adenoma (*n* = 3) versus FTC (*n* = 2) and oxyphilic cell carcinoma (*n* = 4) [55]. In 2020, Oliveira et al. also assessed the ability of RS to discriminate MTC. Analysis of 117 MTC cells, compared to 127 benign and 121 PTC (classic variant) cells, revealed a prominent Raman peak at 1003 cm^−1^ (phenylalanine), significantly overexpressed in MTC (+84% vs. benign; +226% vs. PTC). Finally, classification models achieved accuracies of 97% and 99% for distinguishing MTC from benign and PTC cells, respectively [56].

The only RS study conducted in the context of a preoperative assessment with FNA on thyroid nodules in vivo was performed by Palermo et al. in 2022 [57]. This study involved 123 patients with cytological diagnoses classified as indeterminate or suspicious for malignancy (Bethesda III–VI) and who were candidates for surgery. RS data were compared with cytological findings and the final histological diagnosis, assessing their accuracy in distinguishing between benign and malignant tissue, without differentiating among the various histotypes of thyroid carcinoma. RS demonstrated an overall specificity of 86.8% in predicting nodule malignancy, which increased when selecting nodules classified as Bethesda III and IV with high-risk US features, showing specificities of 87.5% and 100%, respectively. Furthermore, RS proved to be a reliable “rule out” test for Bethesda III nodules (NPV of 92%) and a “rule in” test for Bethesda IV nodules (positive predictive value of 93.3%). The underlying cause of false negatives (17.8% in total) was identified as the issue of reduced or inadequate cellularity of the sample. False positives (overall 4.06%) were instead attributed to the presence of FAs, which, although not malignant, exhibit altered expression of biomolecules such as galectin-3 or HBME [58]. This is in line with what is known about genetic mutations (e.g., RAS) that are already present in benign lesions [57].

In conclusion, both FT-IR and RS hold promising potential for medical applications. These techniques are relatively simple to perform, require only minimal sample quantities, and do not involve complex preparation procedures. As with conventional cytology, spectroscopic analysis may be affected by sampling errors during FNA. This limitation has not been extensively addressed in the literature. As previously mentioned, Schultz et al. proposed that spectroscopy could detect metabolic alterations even in low-cellularity samples [52], though this remains to be validated. The main limitations of these methodologies include the availability of the instrumentation and the need to standardize instrumental parameters and cytological sample selection across multiple centers. To enable their routine use in clinical settings, the development of a comprehensive spectral profiling database and the creation of a diagnostic algorithm suitable for outpatient applications would be required.

To sum up, FNA metabolomic evaluation, through the described techniques, could provide an excellent ancillary method to improve the accuracy of cytopathological evaluation and better define a patient’s management. While the evaluation of FNA is already non-invasive and cost-effective, other samples, such as blood and urine, have been used more recently to potentially develop high-throughput auxiliary tools for screening for thyroid malignancies. Indeed, blood samples and urine are able to convey more information on the general metabolism of the body, potentially providing a more comprehensive system-level picture compared to FNA. Abooshahab and colleagues, for instance, explored the potential of Untargeted gas chromatography quadrupole-MS in differentiating between benign and malignant lesions on blood samples. Remarkably, they found profound differences in the metabolism between thyroid nodule patients and healthy subjects, with significant alterations in metabolite levels associated with sucrose and amino acid metabolism, tricarboxylic acid cycle, fatty acid metabolism, and purine and pyrimidine metabolism [59]. Further innovation was represented by the application of gold-doped zirconium-based metal−organic framework nanostructures for metabolic screening and profiling of thyroid diseases using urine samples. With the use of different machine learning techniques and the creation of a diagnostic panel, Chen and colleagues achieved a remarkable diagnostic accuracy of 98.6% for discriminating thyroid malignancies from low-risk thyroid nodules [60]. The integration of non-invasive blood or urine testing with US evaluation can potentially enhance the reliability of the single non-invasive diagnostic methods for screening of malignancies. This ultimately aims at preventing unnecessary invasive procedures and overtreatment in patients with benign thyroid nodules.

## 5. Conclusions

**Figure 4:**
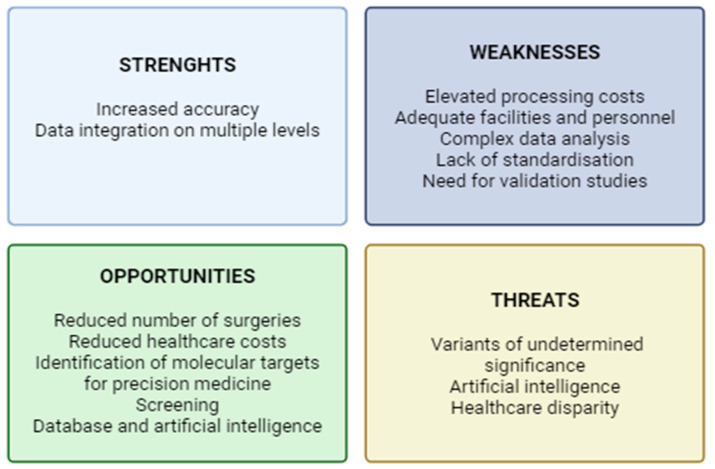
SWOT analysis of the multi-omic approach for the management of nodules of undetermined cytology (created using BioRender.com) (accessed on 20 April 2025).

The current literature on the techniques described suggests that they represent a potential ancillary tool to optimize the management of patients presenting with undetermined thyroid nodules. To produce an effective synthesis of current evidence, we applied the SWOT (strengths, weaknesses, opportunities, and threats) analysis, a method introduced from the business world and recently adapted to medical research [61] (Figure 4).

Despite the lack of literature describing the combination of multiple techniques, a multi-omics approach, through the integration of genomic, proteomic, and metabolomic profiles with clinical data, could potentially maximize the diagnostic accuracy, thus reducing the number of unnecessary invasive procedures and healthcare costs. Nevertheless, the multi-omic approach requires funding, advanced facilities, and qualified personnel; it also produces huge amounts of data that require complex elaboration and appropriate storage. Moreover, given the recent introduction of these techniques, there is still a lack of standardization and huge variability between different laboratories and heterogeneity of the techniques used.

In the future, a comprehensive analysis through the different techniques could also help elucidate various pathogenetic mechanisms and define new molecular targets for therapy. These techniques, as presented above, could also be applied to samples such as blood or urine, thus representing a valuable tool for potential screening for thyroid malignancies. The huge amount of data from multi-omic analyses could be used to generate databases and processed through machine learning as a supporting decisional tool for physicians. These tools, however, are to be exploited with caution; indeed, the presence of distinct genetic variants, proteomic or metabolomic signatures, is not necessarily implied in the pathogenetic mechanisms of malignancy, and in-depth validation studies are therefore required. The identification of a variant of undetermined significance can, nonetheless, generate doubt in the physician and anxiety in a patient, and lead to overdiagnosis and potential overtreatment. There is also the risk, for the clinician, to exclusively rely on artificial intelligence, overlooking important clinical details. The use of these technological advances will also be restricted to some rich areas, thus increasing the disparity in healthcare privileges.

## Data Availability

Not applicable.

## Abbreviations

The following abbreviations are used in this manuscript:

ATC	Anaplastic thyroid carcinoma
CCD	Charge-coupled device
DESI-MS	Desorption electrospray ionization mass spectrometry
ELISA	Enzyme-Linked Immunosorbent Assay
FA	Follicular adenoma
FNA	Fine needle aspiration
FT-IR	Fourier Transform Infrared
FTC	Follicular thyroid cancer
GC	Genomic Classifier
GC−TOFMS	Gas chromatography−time of-flight mass spectrometry
IR	Infrared
LDA	Linear discriminant analysis
MS	Mass spectrometry
MALDI	Matrix-assisted laser desorption
MALDI-IMS	Matrix-assisted laser desorption ionization/imaging mass spectrometry
MALDI-TOF/MS	Matrix-assisted laser desorption ionization time-of-flight/mass spectrometry
NGS	Next-generation sequencing
NIFTP	Non-invasive follicular thyroid neoplasm with papillary-like nuclear features
PDTC	Poorly differentiated thyroid carcinoma
PPV	Positive predictive value
PNV	Negative predictive value
PI	Phosphatidylinositol
PTC	Papillary thyroid cancer
RS	Raman Spectroscopy
TA	Thyroid adenoma
UPLC−QTOFMS	Ultraperformance liquid chromatography−quadrupole time-of-flight mass spectrometry
US	Ultrasound
WB	Western blot
